# Comparison of Orbital Atherectomy and Rotational Atherectomy in Calcified Left Main Disease: Short-Term Outcomes

**DOI:** 10.3390/jcm12124025

**Published:** 2023-06-13

**Authors:** Piotr Rola, Jan Jakub Kulczycki, Mateusz Barycki, Szymon Włodarczak, Łukasz Furtan, Michalina Kędzierska, Katarzyna Giniewicz, Adrian Doroszko, Maciej Lesiak, Adrian Włodarczak

**Affiliations:** 1 Faculty of Health Sciences and Physical Culture, Witelon Collegium State University, 59-220 Legnica, Poland; wlodarczak.adrian@gmail.com; 2 Department of Cardiology, Provincial Specialized Hospital, 59-220 Legnica, Poland; mateusz.barycki@gmail.com (M.B.); lukas.furtan@gmail.com (Ł.F.); 3 Department of Cardiology, The Copper Health Centre (MCZ), 59-300 Lubin, Poland; jan.jakub.kulczycki@gmail.com (J.J.K.); wlodarczak.szy@gmail.com (S.W.); 4 Faculty of Medicine, Wroclaw Medical University, 50-556 Wroclaw, Poland; kedzierska.michalinaa@gmail.com; 5 Independent Researcher, 50-556 Wroclaw, Poland; katarzyna@giniewicz.it; 6 Clinical Department of Internal Medicine and Occupational Diseases, Hypertension and Clinical Oncology, Faculty of Medicine, Wroclaw Medical University, 50-556 Wroclaw, Poland; adrian.doroszko@gmail.com; 7 1st Department of Cardiology, University of Medical Sciences, 61-848 Poznan, Poland; maciej.lesiak@skpp.edu.pl

**Keywords:** orbital atherectomy, rotational atherectomy, left main disease, percutaneous coronary intervention, calcified lesions

## Abstract

Background: Coronary calcifications, particularly in left main disease (LMD), are independently associated with adverse outcomes of percutaneous coronary intervention (PCI). Adequate lesion preparation is pivotal to achieve favorable short- and long-term outcomes. Rotational atherectomy devices have been used in contemporary practice to obtain adequate preparation of the calcified lesions. Recently, novel orbital atherectomy (OA) devices have been introduced to clinical practice to facilitate the preparation of the lesion. The objective of this study is to compare the short-term safety and efficacy of orbital and rotational atherectomy for LMD. Methods: we retrospectively evaluated a total of 55 consecutive patients who underwent the LM PCI supported by either OA or RA. Results: The OA group consisted of 25 patients with a median SYNTAX Score of 28 (26–36). The Rota group consisted of 30 patients with a median SYNTAX Score of 28 (26–33.1) There were no statistical differences in MACCE between the RA and OA subpopulations when recorded in-hospital (6.7% vs. 10.3% *p* = 0.619) as well as in a 1-month follow-up after the procedure (12% vs. 16.6% *p* = 0.261). Conclusion: OA and RA seem to be similarly safe and effective strategies for preparating the lesion in the high-risk population with calcified LMD.

## 1. Introduction

Despite the fact that significant left main disease (LMD) is a rather uncommon finding in patients with stable [1] or unstable [2] coronary artery disease (CAD), a significant narrowing of the LM is related to a potentially large area of ischemia leading to high-risk adverse cardiac events. The traditional approach for the management of LMD includes coronary artery bypass grafting (CABG), particularly if additional risk factors of an unfavorable outcome of percutaneous coronary intervention (PCI) occur. Coronary calcifications are independently associated with adverse outcomes of PCI [3] and their correlation with LMD remains one of the most challenging areas. Indeed, calcifications increase the probability of acute and long-term stent failure [4] therefore adequate lesion preparation is a crucial part of the therapeutic process [5].

However, continuous improvement in terms of PCI technique, supported by the introduction to clinical practice novel devices, which allows adequate lesion preparation, is changing the old paradigm in relation to the indisputable need for surgical treatment of calcified LM lesions. As a result of population aging along with constantly increasing comorbidity despite the presence of an unfavorable risk factor including calcifications of a high SYNTAX Score, the PCI often provides the only available revascularization strategy.

Rotational atherectomy (RA) is the first and among the most valued of the debulking methods introduced to clinical practice. The mechanism of action is based on the high-speed rotation (140,000 to 180,000 rpm) of a diamond-encrusted elliptical burr. The burr gradually ablates calcified plaque as it is advanced across the lesion on a dedicated guidewire. Despite the high prevalence of coronary calcification in the CAD and even though this system has been on the market for over 3 decades (with the upgrade to Rota-Pro), the utilization of RA in select centers varies from 1% to 5% [6,7]. RA is technically challenging and patients undergoing RA may experience several complications (perforation, side branch loss, burr trapping, and slow flow/no-reflow phenomenon). Although RA has matured as a therapeutic tool, data focused on RA in the LM are scarce, mostly out of date, and still need reevaluation including prospective observation [8,9].

Recently, novel atherectomy devices such as the Diamondback 360 Coronary Orbital Atherectomy (OA) (Cardiovascular Systems Inc., Saint Paul, MN, USA), which facilitate lesion preparation, have been introduced to clinical practice. OA is a debulking device with an eccentrically mounted single-size diamond-coated crown (1.25 mm). The device uses centrifugal force to orbit, allowing for athero-ablation of calcified plaque. The unique mechanism of action of OA as compared to RA (bi-directional burr passage with additional elliptical propagation) along with the procedural support provided by the infusion of ViperSlide lubricant fluid has been postulated to reduce complications [10]. Similarly to RA, the data focused on the utility of OA in terms of LMD are scarce, and limited to a few series of case studies [11].

Even though the OA is a novel device that is not as widely used in clinical practice as RA, two meta-analyses comparing both of them have recently been published. The results of both studies regarding the prevalence of MACE are compatible and indicate no significant differences [12,13]. However, there are some inconsistencies in the analysis of secondary outcomes in both studies. It should be emphasized that neither the mentioned meta-analyses nor any other studies conducted so far have focused on comparing RA and OA in terms of left main disease (LMD). Taking into account the fact that a large number of recently published studies [14,15] have demonstrated relevant differences in terms of physiology, technical aspects of the PCI procedure, and clinical outcomes between the LM and non-LM lesions, we designed this study to evaluate the safety and efficiency of RA and OA in terms of LMD.

## 2. Materials and Methods

### 2.1. A Study Design and Population

The study population consisted of consecutive patients undergoing left main PCI requiring lesion preparation with rotational or orbital atherectomy due to severe calcification. The study is retrospective and observational. All PCI procedures were performed between January 2014 and February 2023 in two cooperative, high-volume PCI cardiac centers in Lower Silesia in Poland (Provincial Specialized Hospital in Legnica and The Copper Health Centre (MCZ)). The decision to perform the PCI was either based on the judgment of the local Heart Team or on specific clinical indications (persistent ischemia or lack of consent for the alternative therapies). All patients were thoroughly informed about alternative therapeutic options and procedural risks. This study had been accepted by the local ethical board (nr 04/BOBD/2022). After accepting the proposed percutaneous mode of treatment, they provided written informed consent for the procedure. Exclusion criteria related to lesion anatomy or complexity did not exist. 

### 2.2. PCI Procedures

All procedural features and clinical decisions, including the use of OA or RA; vascular access point; the sizing of the guiding catheter, burr sizing, ablation speed; number and time of burr passages throughout the lesion; the use of left ventricular supporting devices, intravascular imaging guidance (OCT/IVUS), and inhibitors of glycoprotein IIb/IIIa or catecholamines administration; and stenting technique, were determined at the operators’ discretion, and—when applicable—precisely followed the current ESC guidelines.

Rotational procedures, similar to rotational atherectomy device technology, were performed by experienced interventional cardiologist operators who had previously undergone dedicated training and courses. All the RA procedures were performed in accordance with contemporary clinical practice and in line with the expert consensus document [16].

Similarly, orbital atherectomy PCI was performed by experienced PCI operators who underwent training and obtained the necessary certificates to perform the OA procedures. All OA procedures were performed in accordance with contemporary clinical practice [10,17].

### 2.3. Study Endpoints

The main endpoint of the study was the occurrence of in-hospital major adverse cardiac and cerebrovascular events (MACCE). MACCE were defined as death, myocardial infarction, cerebrovascular events, episodes of target vessel revascularization, and probable/definite stent thrombosis. The secondary endpoints included the occurrence of MACCE after 1 month post-procedure, all kinds of revascularization procedures, cerebrovascular episodes, and stent restenosis. Target vessel revascularization was defined as any repeat intervention (percutaneous or surgical bypass) on any target vessel segment, including the target lesion [18]. Myocardial infarction was defined according to The Fourth Universal Definition of Myocardial Infarction [19]. Stent thrombosis and restenosis definitions were based on The Academic Research Consortium-2 consensus [18]. 

### 2.4. Statistical Analysis

Continuous variables with a normal distribution were characterized using their mean and standard deviation; otherwise, median and interquartile ranges were used. Frequencies were used for categorical variables. Differences between the means were evaluated using the Student’s *t*-test or the Mann–Whitney U test, based on the distribution of the variables and the variety of variances. For this purpose, the Shapiro–Wilk test and Levene’s test were used. The Fisher’s exact test was used for categorical variables. The statistical significance level was set at *p* < 0.05. R language version 4.0.4 was used to perform all the analyses.

## 3. Results

The study population consisted of 55 consecutive patients who underwent the left main PCI facilitated by the use of a rotational or orbital atherectomy device. 

**Figure 1:**
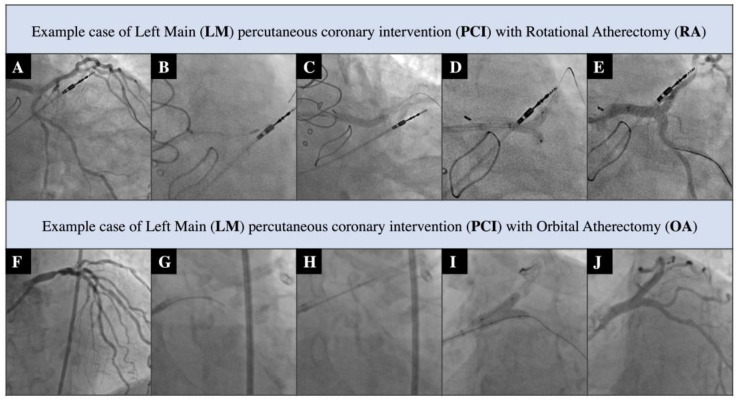
Example LM PCI. Abbreviations: (**A**) Distal LM critical stenosis with severe calcifications; (**B**) RA with burr 1.75 mm; (**C**) LM/LAD stenting with DES 4.0 × 20 mm; (**D**) LM/LAD/Cx kissing balloons with non-compliant 3.5 mm/3.0 mm balloons; (**E**) Final post-PCI angiogram; (**F**) Distal LM critical stenosis with severe calcifications; (**G**) Burr passage from Cx towards LM; (**H**) Burr passage from LAD towards LM; (**I**) LM/LAD/Cx kissing balloons performed with NC 3.5 mm balloon catheters; (**J**) Final post-PCI angiogram.

Exemplary PCI procedures are shown in Figure 1. 

Baseline clinical and demographic characteristics of the two study groups are detailed in Table 1.

The OA group consisted of 25 patients, predominantly male (68%). The vast majority of PCI procedures were performed in the acute coronary syndrome (ACS) setting (76%). The mean age was 68.4 ± 7.9 years. 

Similarly, the majority of subjects in the RA group (n-30) were male (70.0%), with a mean age of 70.4 ± 8.9 years. A relatively high prevalence of ACS cases (56.7%) was also observed in this study group, and there was no difference regarding the ACS and CCS setting between groups. A high prevalence of cardiovascular risk factors was observed in both cohorts, with only hyperlipidemia showing a significant difference (96% vs. 70%, respectively, *p* = 0.015). 

Significant differences between study cohorts in terms of initial anatomical advancement of CAD were observed—the OA group had a significantly higher SYNTAX II PCI Score than the RA group (22.9 vs. 10.8; respectively, *p* = 0.005), as well as a significantly higher SYNTAX II CABG score (44.3 vs. 34.2; respectively, *p* = 0.016). Analysis of procedural features in both parts of the study revealed there was a higher prevalence of radial access (80% vs. 50%, respectively, *p* = 0.026) and a higher ratio of smaller (6F) access points (64% vs. 10%, respectively, *p* = 0.015) in the OA cohort. The overall active burr ablation time was also higher in the OA group (225 vs. 180, respectively, *p* = 0.015). On the other hand, in the RA group we observed a higher median of total stent length implanted during the index procedure (75 mm vs. 60 mm, respectively, *p* = 0.018). Subjects in the OA group were more prone to receive intravascular guidance during PCI compared to the RA cohort (70% vs. 10%, respectively, *p* = 0.001) Figure 1 presents example cases of RA and OA LM PCI. Table 2 contains baseline procedural features.

**Figure 2:**
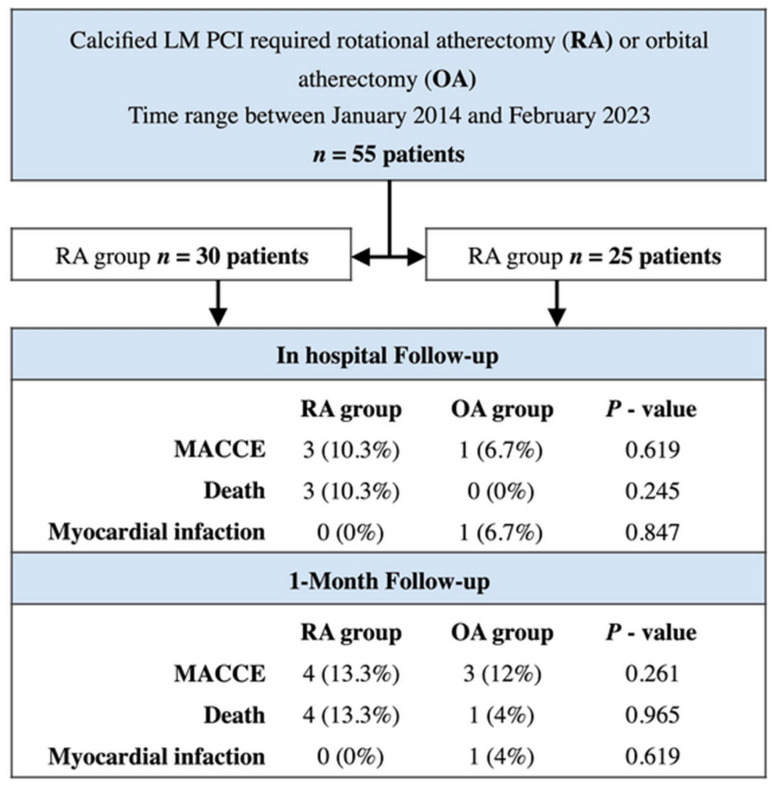
Study flow-chart and outcomes (central illustration/graphical abstract). Abbreviations: MACCE—Major adverse cerebrovascular and cardiac events.

There were no significant differences in the primary and secondary outcomes at 1 month after the procedure. In the OA cohort, we observed one periprocedural MI (acute Cx occlusion after LM/LAD stenting). In addition, one patient died within 21 days of the procedure due to multiorgan dysfunction and another patient 15 days following discharge experienced a TIA. There were three in-hospital deaths in the RA group. One death was periprocedural—a patient with pre-hospital cardiac arrest. The procedure was performed with the support of the Lucas CPR device (Stryker Medical, Portage, MI, USA). Two deaths were post-procedural (patients were transferred to the ICU after PCI because of multiorgan dysfunction; deaths were observed at 10 days and 17 days after the PCI procedure); we noted one unexplained death 17 days after discharge in a patient with multiple comorbidities and alcohol abuse. Significant differences in planned revascularization (any revascularization) between the study groups (44% vs. 6.9%, respectively, *p* = 0.013) reflect organizational rather than clinical issues in cardiac care. They are likely to disappear as the clinical follow-up is extended. Study outcomes are presented in Figure 2, and Table 3. 

## 4. Discussion

To our knowledge, this study is the first to compare the short-term safety and efficacy of orbital atherectomy (OA) devices with conventional rotational atherectomy (RA) for left main disease (LMD). 

The key findings of this study are as follows:

− In the high-risk study cohort, orbital atherectomy demonstrated a good short-term safety profile that was comparable to that of conventional rotational atherectomy.
− There were no significant differences in primary or secondary outcomes between the two debulking devices.

Patients with calcified LMD are typically considered a high-risk population. Current guidelines [20] strongly support CABG as the preferred method of revascularization, especially in subpopulations with a high degree of anatomical complexity of CAD (SYNTAX Score greater than 22). Since the mean syntax score in both study cohorts reached 28, the population of our study should be considered a high-risk population for PCI. The potential technical difficulties during a PCI procedure, together with the large area of ischemic territory determine that any periprocedural complications during the PCI can become life-threatening.

Slow/flow phenomena and vessel perforation are two major concerns regarding procedural complications during rotational atherectomy. The established risk factors for peripheral flow abnormalities during atherectomy procedures include the time of athero-ablation and the size of the burr [21,22,23,24]. Surprisingly, despite the significantly longer burr time used in the OA cohort (225 s vs. 180 s), no significant difference in slow/flow phenomena was observed. In fact, the overall percentage of distal flow impairment was lower in the OA group (4% vs. 6%). The low prevalence of this adverse event had been previously described in other OA studies [25,26,27,28]. This fact is likely related to the centrifugal burr mechanism of action along with the relatively small crown diameter (1.25 mm). Orbital movement through a lesion allows the maintenance of continuous blood flow through the vessel along with debris reduction when compared to the classic RA [29,30]. 

Vascular access characteristics were another important factor influencing the safety of OA. In our study, we observed a significantly higher rate of radial access [80% vs. 50%, respectively, *p*= 0.026], along with a smaller catheter size [64% vs. 10%, respectively, *p* = 0.015]. This has been shown to have a strong impact on procedural safety [31,32] and had been previously described in other studies comparing RA vs. OA in non-LM lesions [28,33]. These clinical advantages of the OA PCI procedure are achieved by the lower profile (1.25 mm) orbital atherectomy burr. The acceleration of a crown speed allows the treatment of a vessel with a larger lumen (1,200,000 rpm allows the treatment of vessels with a lumen greater than 3.0 mm) without the need to upsize the burr, as is required in the case of classical rotational atherectomy. This may be particularly useful in the case of high-grade calcified lesions in large vessels, which certainly include LM. While this benefit appears to be significant, large, randomized studies are needed to validate it. Although OA interacts with the deep calcium deposits in some rare cases, subsidiary modification is required [34].

Given the complexity of the disease and the high prevalence of ACS cases along with the co-morbidities of the patients recruited in this study, the short-term clinical outcomes (MACE, death, MI, and TLR) in both study groups are equal or superior to many previously reported in the literature [34,35,36,37]. Considering that RA has shown significant improvement in increasing procedural success rates in the treatment of severely calcified coronary lesions [38] results of our pilot study suggest that OA in high-risk populations with LMD showed a similar level of safety and efficiency. 

Recently, the potential armamentarium used for the preparation of calcified lesions has expanded and novel devices have been used in clinical practice [39,40,41,42,43,44]. A presented study suggests that OA may be useful in terms of LMD, however, we still see a strong need for high-volume long-term randomized trials comparing available debulking methods (OA, RA, S-IVL, laser atherectomy; cutting/scoring balloons) to assess their effectiveness. 

### Limitations

The observational and retrospective design is the major limitation of this study. In addition, the decision to use RA or OA was left to the discretion of the operators. Due to the novelty of OA and its availability in our cardiac center, the recruitment timeframe is not identical in both cohorts, which may partially affect the results. Additional intravascular imaging was not mandatory during PCI procedures, so its prevalence is relatively low. Moreover, the study population is relatively small and probably underpowered, but it should be noted that the study includes patients with the most advanced CAD and the number of patients recruited is analogous to previously published studies. The small number of participants and lack of external core laboratory analysis, particularly with respect to intravascular imaging, prevented the selection of potential demographic, clinical, or anatomic risk factors for adverse outcomes.

## 5. Conclusions

In patients with calcified left main disease the orbital atherectomy and rotational atherectomy appear to be safe and effective with acceptable in-hospital and short-term outcomes considering the severity of disease and lesion characteristics.

## Figures and Tables

**Table 1:** The baseline clinical characteristics of both study groups.

	Rotational Atherectomy (RA) N-30	Orbital Atherectomy (OA) N-25	*p*-Value
Age	70.4 ± 8.9	68.4 ± 7.9	0.398
Gender male (ratio)	21 (70%)	17 (68.0%)	0.584
Stable angina	13 (43.3%)	6 (24%)	0.163
Unstable angina	7 (23.3%)	3 (12%)	0.318
NSTEMI	9 (30.0%)	16 (64%)	0.106
STEMI	1 (3.4%)	0 (0%)	1
Diabetes mellitus	16 (53.3%)	11 (44%)	0.591
Chronic heart failure	10 (34.5%)	14 (56%)	0.170
Hypertension	26 (86.7%)	24 (96%)	0.362
Hyperlipidemia	21 (70%)	24 (96%)	0.015
Atrial Fibrillation	6 (20%)	6 (24%)	1
History of PCI	16 (53.3%)	11 (44%)	0.591
History of MI	14 (46.7%)	12 (48%)	1
History of CABG	6 (20%)	2 (8%)	0.269
COPD	8 (26.7%)	10 (40%)	0.389
CKD	13 (43.3%)	6 (24%)	0. 162

Abbreviations: NSTEMI—non-ST-Elevation Myocardial Infraction; STEMI—ST-Elevation Myocardial Infraction; PCI—percutaneous coronary intervention; MI—Myocardial Infraction; CABG—coronary artery bypass grafting; COPD—Chronic Obstructive Pulmonary Disease; CKD—Chronic Kidney Disease.

**Table 2:** The baseline procedural features.

	Rotational Atherectomy (RA) N-30	Orbital Atherectomy (OA) N-25	*p*-Value
SYNTAX I Score	28 (26–33.1)	28 (26–36)	0.874
SYNTAX II—PCI Score	36.2 ± 8.6	45.3 ± 13.3	0.005
SYNTAX II PCI four-year mortality	10.8 (7.7–17.8)	22.9 (9.3–41.1)	0.009
Syntax II—CABG Score	34.2 (28.2–43.6)	44.3 (37.5–49.3)	0.010
Syntax II CABG four-year mortality	10.3 (6.1–20.5)	21 (12–30)	0.016
Radial Access	15 (50%)	20 (80.0%)	0.026
6F Guide Catheter	3 (10%)	16 (64%)	0.015
7F or larger Guide Catheter	27 (90%)	14 (56%)	0.015
Total ablation time (s)	180 (140–190)	225 (200–300)	0.002
Average number of stents	2 (2–2.75)	2 (1–2)	0.072
Total stent length per procedure (mm)	75 (50–90.3)	60 (30–72)	0.018
Post-dilation—POT	29 (96.7%)	25 (100%)	0.494
Intravascular Guidance	3 (10%)	18 (72%)	0.001
Perforation	3 (10%)	1 (4%)	0.617
Slow/No-flow phenomenon	2 (6.0%)	1 (4%)	1
Administration of catecholamines	3 (10%)	3 (12%)	1
Acetylsalicylic Acid	30 (100%)	15 (100%)	1
Clopidogrel	19 (63.3%)	16 (64%)	1
Ticagrelor	10 (33.3%)	7 (28%)	0.773

Abbreviations: PCI—percutaneous coronary intervention; CABG—coronary artery bypass grafting; POT—proximal optimization technique.

**Table 3:** Clinical follow-up data of both groups.

	Rotational Atherectomy (RA) N-30	Orbital Atherectomy (OA) N-25	*p*-Value
In-hospital Follow-up			
MACCE	3 (10.3%)	1 (6.7%)	0.619
Death	3 (10.3%)	0 (0%)	0.245
Myocardial infarction	0 (0%)	1 (6.7%)	0.847
Target vessel revascularization	0 (0%)	0 (0%)	0.341
Stent thrombosis	0 (0%)	0 (0%)	0.341
Cerebrovascular episodes	0 (0%)	0 (0%)	-
Stent restenosis	0 (0%)	0 (0%)	-
Need for any revascularization	1 (3.3%)	2 (8%)	0.448
1-Month Follow-up			
MACCE	4 (13.3%)	3 (12%)	0.261
Death	4 (13.3%)	1 (4%)	0.965
Myocardial infarction	0 (0%)	1 (4%)	0.619
Target vessel revascularization	0 (0%)	0 (0%)	-
Stent thrombosis	0 (0%)	0 (0%)	-
Cerebrovascular episodes	0 (0%)	1 (4%)	0.619
Stent restenosis	0 (0%)	0 (0%)	-
Need for any revascularization	4 (6.9%)	11 (44%)	0.013

Abbreviations: MACCE—Major adverse cerebrovascular and cardiac events.

## Data Availability

All data not included in the manuscript are available after contacting the corresponding author in accordance with local legal regulations.
